# Evaluating coral trophic strategies using fatty acid composition and indices

**DOI:** 10.1371/journal.pone.0222327

**Published:** 2019-09-11

**Authors:** Veronica Z. Radice, Michael T. Brett, Brian Fry, Michael D. Fox, Ove Hoegh-Guldberg, Sophie G. Dove

**Affiliations:** 1 Australian Research Council Centre of Excellence for Coral Reef Studies, The University of Queensland, St. Lucia, Queensland, Australia; 2 School of Biological Sciences, The University of Queensland, St. Lucia, Queensland, Australia; 3 Department of Civil and Environmental Engineering, University of Washington, Seattle, Washington, United States of America; 4 Australian Rivers Institute, Griffith University, Nathan, Queensland, Australia; 5 Department of Geology and Geophysics, Woods Hole Oceanographic Institution, Woods Hole, Massachusetts, United States of America; Uniwersytet Warszawski, POLAND

## Abstract

The ecological success of shallow water reef-building corals has been linked to the symbiosis between the coral host and its dinoflagellate symbionts (herein ‘symbionts’). As mixotrophs, symbiotic corals depend on nutrients 1) transferred from their photosynthetic symbionts (autotrophy) and 2) acquired by host feeding on particulate organic resources (heterotrophy). However, coral species differ in the extent to which they depend on heterotrophy for nutrition and these differences are typically poorly defined. Here, a multi-tracer fatty acid approach was used to evaluate the trophic strategies of three species of common reef-building coral (*Galaxea fascicularis*, *Pachyseris speciosa*, and *Pocillopora verrucosa*) whose trophic strategies had previously been identified using carbon stable isotopes. The composition and various indices of fatty acids were compared to examine the relative contribution of symbiont autotrophy and host heterotrophy in coral energy acquisition. A linear discriminant analysis (LDA) was used to estimate the contribution of polyunsaturated fatty acids (PUFA) derived from various potential sources to the coral hosts. The total fatty acid composition and fatty acid indices revealed differences between the more heterotrophic (*P*. *verrucosa*) and more autotrophic (*P*. *speciosa*) coral hosts, with the coral host *G*. *fascicularis* showing overlap with the other two species and greater variability overall. For the more heterotrophic *P*. *verrucosa*, the fatty acid indices and LDA results both indicated a greater proportion of copepod-derived fatty acids compared to the other coral species. Overall, the LDA estimated that PUFA derived from particulate resources (e.g., copepods and diatoms) comprised a greater proportion of coral host PUFA in contrast to the lower proportion of symbiont-derived PUFA. These estimates provide insight into the importance of heterotrophy in coral nutrition, especially in productive reef systems. The study supports carbon stable isotope results and demonstrates the utility of fatty acid analyses for exploring the trophic strategies of reef-building corals.

## 2. Introduction

The evolutionary success of symbiotic reef-building corals within typically nutrient-poor oceans is attributed to their symbiosis with dinoflagellates [1]. As mixotrophic organisms, symbiotic reef-building corals have different trophic strategies that support their distribution across a wide range of environmental conditions [2]. Under conditions of reduced light (e.g., shaded areas or deep reefs) and/or in reefs with high food availability (turbid reefs), the translocation of photosynthetically-acquired carbon may be reduced and external food sources can be an important component of coral nutrition [3,4]. Symbiotic corals obtain nutrients from their photosynthetic dinoflagellate endosymbionts (herein ‘symbionts’; autotrophy) and from feeding on particulate resources in the water column (heterotrophy), including bacteria, detritus, flagellates, phytoplankton, and zooplankton [5]. Moreover, heterotrophy can have a positive feedback on symbiont autotrophy [6]. The low bioavailability and concentration of dissolved organic matter makes it a minor potential component of coral nutrition in contrast to nutrients being i) transferred from symbionts and ii) assimilated from heterotrophic feeding on particulates [5,7]. The role of heterotrophy greatly varies in relation to environmental factors and species-specific feeding rates, with heterotrophic carbon accounting for a significant, but highly variable, proportion of daily requirements in some coral species [8]. Indeed, it is challenging to estimate the relative importance of heterotrophy because laboratory-based studies cannot account for *in situ* environmental effects and calculations require a series of assumptions. The ability of corals to increase heterotrophy and assimilate lipids (as estimated from feeding rates and carbon isotope ratios) is species specific [8,9]. Lipids are important for energy storage and comprise a major constituent of coral carbon content and overall dry weight, with variation in total lipids among species [10].

As the major component isolated from coral total lipids [11,12], fatty acids (up to 73% of total lipids) reflect patterns in coral nutrition and have been used in various ways to assess trophic strategies of corals [13]. Fatty acids are essential for energy use and storage, cell membrane structure, and gene regulation [14]. Focusing on individual fatty acids in attempt to divide the coral holobiont into symbiont (plant) versus coral (animal) synthesized groups as an indication of autotrophic versus heterotrophic contributions must be carefully considered because symbiotic corals are basal animals that have some plant-like capacities [15]. Due to the carbon exchange between coral hosts and symbionts [16], the fatty acid composition of cultured symbionts differ from those of coral-associated symbionts [17,18]. Further, as dinoflagellates can be mixotrophic, coral-associated symbionts are not inherently limited to autotrophy and some symbionts in the family Symbiodiniaceae (formerly *Symbiodinium* spp.) have been shown to shift to parasitism under some conditions [19–21]. Much of the literature on coral fatty acids has assumed that corals, as animals, lack certain desaturases (i.e., Δ12 and Δ15) required for the synthesis of metabolically important PUFA. However, recent work has shown that cnidaria, including scleractinian corals, have genes for *de novo* biosynthesis of n-3 PUFA and desaturase activity has been functionally characterized for a variety of aquatic invertebrates [22–24]. Indeed, certain long-chain fatty acids (i.e., C_22_) appear to be host-derived rather than symbiont-derived [25]. In addition to the transfer of fatty acids from the symbionts to the coral host [26], the coral host can also transfer PUFA to their symbionts [27]. The ability of both symbiotic partners to synthesize and transfer fatty acids, including PUFA, complicates the delineation between each partner’s biosynthetic pathways and hinders the use of individual fatty acids as trophic biomarkers. Indeed, there has been discrepancy regarding the composition and utility of “autotrophic” fatty acid markers produced by symbionts. Despite these challenges, the application of different metrics for analyzing fatty acids may assist in discerning the differential contributions of autotrophy to heterotrophy, especially when combined with knowledge of key food source fatty acid composition [28].

Fatty acids are good markers for chemotaxonomy that can distinguish between plankton groups [29,30], scleractinian coral genera, and symbiotic and asymbiotic corals [31,32]. Fatty acid biomarkers provide an integrated record of dietary input over time, reflecting the assimilation and translocation of nutrients [33]. Specifically, PUFA composition can resolve scleractinian coral genera and has been used to evaluate potential differences in trophic strategies [28]. However, experimental studies have shown variable effects of feeding on coral host fatty acid composition [11,12,34]. Fatty acid indices are commonly used in aquatic ecology to evaluate trophic strategies, but have yet to be applied in the context of trophic ecology of corals [35]. First, the ratio of fatty acids 18:1n-7 to 18:1n-9 is used to evaluate relative herbivory versus carnivory because the former is produced by the elongation of 16:1n-7 that is likely derived in substantial quantities from photosynthetic organisms (e.g., formerly *Symbiodinium* spp.) and the latter is a major fatty acid in most marine animals [36–38]. Although this ratio is not an explicit indicator, recent work showing different proportions of 16:1n-7 in cnidarians hosting diverse Symbiodiniaceae supports the idea that 16:1n-7 may reflect photosynthetically-derived input [39]. Therefore, we also consider a correlated fatty acid index to characterize the ratio of typical photosynthetic input (sum of fatty acids 16:1n-7 and 18:1n-7) to typical animal dietary input (sum of fatty acids 18:1n-9, 20:1n-9, and 22:1n-11) [40]. Another index, the sum of long-chain monounsaturated fatty acids (LC-MUFA = Σ20:1 and Σ22:1), is used to evaluate potential feeding on copepods since herbivorous marine copepods, including tropical copepods, can have elevated concentrations of LC-MUFA [30,35,41]. Tropical symbiotic reef-building corals (e.g., *Goniopora*) and hydrocorals (e.g., *Millepora*), which can feed extensively on plankton, have been shown to have elevated proportions of LC-MUFA (~5–6% of total fatty acids) [42]. Further, symbiotic reef-building coral hosts experimentally fed with particulate food sources show higher proportions of LC-MUFA than starved corals while LC-MUFA are often negligible in symbionts [11,43]. The LC-MUFA index has been previously applied to cold-water corals and subtropical symbiotic jellyfish but has yet to be considered for tropical symbiotic corals [44,45]. Despite the taxonomic diversity of planktonic food sources in reef systems, experimental feeding studies on tropical scleractinian corals typically focus on only one potential particulate food source; most often being *Artemia* nauplii, which are not a natural prey item for corals. Ideally, trophic studies should account for environmental factors that may affect prey and dissolved nutrient composition and availability in reefs, including seasonality if applicable [46,47]. However, temporal and spatial information on plankton community dynamics in tropical coral reef ecosystems remains limited [48,49], especially in relation to the trophic ecology of reef-building corals.

Here, we evaluate the utility of fatty acid biomarkers in characterizing coral trophic strategies by investigating the coral fatty acid composition of three species of reef-building corals (*Galaxea fascicularis*, *Pachyseris speciosa*, and *Pocillopora verrucosa*) and their associated symbionts. Because dietary fatty acids can be modified in consumers, this study focused on corals for which prior knowledge of species-specific trophic strategies is available [50]. The fatty acid composition of coral hosts and symbionts from reefs at different depths (10 and 30 m) were investigated because depth-related factors may affect the fatty acid composition of corals [42,51] and previous research in the same reef system showed differential patterns of carbon stable isotopes in *G*. *fascicularis* over depth. We also provide a comprehensive evaluation of the trophic strategies of symbiotic corals by 1) examining the composition of multiple tracers (i.e., total [30] fatty acids, PUFA composition), 2) applying three established fatty acid indices in the novel context of coral trophic ecology, and 3) estimating the contribution of various sources of PUFA (symbionts and diverse plankton groups) to each coral host species. Total fatty acid compositions were used to evaluate patterns in coral trophic strategies while the relationship between host and symbiont proportions of individual fatty acids was used to investigate nutritional cycling between the symbiotic partners. Established fatty acid indices were applied in a novel context to examine relative autotrophy and heterotrophy among the coral species. Two different fatty acid indices were used to evaluate nutritional input from photosynthetic- versus animal-derived sources while another index tested for potential feeding on copepods. We hypothesized that the relatively more autotrophic coral *P*. *speciosa* would have a total fatty acid composition that differed from the more heterotrophic coral *P*. *verrucosa*. Because *G*. *fascicularis* can shift its trophic strategy as a function of environmental conditions [52], it was expected that this species would have a fatty acid composition that shared similarities with both other species. It was expected that the different fatty acid indices would show a greater fatty acid input from symbiont autotrophy for *P*. *speciosa* and less for *P*. *verrucosa* and *G*. *fascicularis*. Further, it was hypothesized that the PUFA composition of the more heterotrophic coral *P*. *verrucosa* would be more similar to the plankton prey groups compared to the other two coral species. Using multiple biochemical tracers, our study evaluates trophic strategies among three morphologically diverse species of coral and considers potential sources for coral heterotrophic feeding.

## 3. Methods

### 3.1 Sample collection

Fragments of three species of coral (*Galaxea fascicularis*, *Pachyseris speciosa*, and *Pocillopora verrucosa*) were collected by divers from shallow (10 m depth) and deep (30 m depth) reefs in the central Maldives, Indian Ocean, in March-April 2017 (S1 Table). In this study, coral fragments were randomly sampled for fatty acid analyses following a previous study across the central Maldives. This study showed that coral trophic strategies, as characterized by carbon stable isotopes, were not affected by reef site [50]. Coral fragments were rinsed with filtered (0.4 μm) seawater and immediately frozen until analysis. Isolation of the host fraction followed the methodology of Radice et al. [50] where tissue was separated from the coral skeleton with filtered (0.22 μm) seawater using a pressurized airbrush. Host tissue was subsequently separated from symbionts by centrifugation (minimum four times at 3,000×g, symbionts resuspended in filtered seawater each time) with homogenates and pellets both acidified to remove any carbonates, and samples freeze-dried prior to fatty acid analysis.

### 3.2 Extraction and analysis of fatty acids

Using a modified Folch method [53] following Taipale et al. [54], total lipids were extracted from freeze-dried coral host (n = 10 per coral species) or symbiont (n = 10 per host species) tissue (mean 5.2 mg ± 0.1 SD). First, 4:2:1 CHCl_3_:MeOH:H_2_O was added to each sample which was subsequently sonicated, mixed, and centrifuged to separate the phases. The organic phase was transferred and total lipids were extracted for a second time, followed by evaporation under N_2_ gas with the remaining lipids dissolved in toluene. Methanolic H_2_SO_4_ was added to resuspend the total lipids, which were methylated when heated in a water bath (90°C for 90 min). Fatty acid methyl esters were extracted twice with n-hexane (evaporated under N_2_ gas) and subsequently dissolved in n-hexane (0.5–1.5 mL) for gas chromatography analysis. The fatty acid methyl esters were analyzed using a Gas Chromatograph coupled with a Flame Ionization Detector (GC-FID, Hewlett Packard HP6890) and an Agilent DB-23 column with an 85 min run time [55]. Fatty acids were identified by comparing retention times and peak area with reference standards (FAME 37; GLC-68D standard Nu Check-Prep; oyster *Ostrea lurida*) and a subset of samples were run on a gas chromatograph equipped with a mass spectrometer (GC-MS QP2010 Plus). The total mass of fatty acids of each coral species and tissue type was quantified using a concentration conversion factor that was generated for different concentrations with a standard curve method. Comparing fatty acid peak area versus mass, the slopes of the linear regressions were consistent among fatty acid standards and the calculated fatty acid concentration was within an average of 1.4% [56]. Using the derived average slope, the mass of each fatty acid per dry weight tissue was calculated using tissue mass, volume hexane, and peak area.

### 3.3 Statistical analyses

Because of the variability of fatty acid composition, data were not transformed in order to avoid falsely inflating the contribution of minor fatty acids [57]. Non-parametric PERMANOVA analyses were used to test whether the relative fatty acid compositions (% total fatty acids) were similar among the hosts and symbionts of the three coral species from shallow and deep reefs (9999 permutations; PRIMER-E version 6 with PERMANOVA add-on, Plymouth, U.K.). PERMDISP, used to test the homogeneity of multivariate dispersion for the significant interaction observed in the PERMANOVA result, showed that the data were homogeneous. To investigate the potential relationship of fatty acid composition between coral host and symbionts, linear regressions were used to compare symbiont and host proportions of individual fatty acids.

To assess coral trophic strategies, established fatty acid indices including 1) the ratio of 18:1n-7 to 18:1n-9 fatty acids, 2) the ratio of photosynthetic- (sum of 16:1n-7 and 18:1n-7) versus animal-derived input (sum of 18:1n-9, 20:1n-9, and 22:1n-11), and 3) the sum of LC-MUFA (20:1n-11, 20:1n-9, 20:1n-7, 22:1n-11, 22:1n-9, and 22:1n-7), a trophic index for herbivorous copepod consumption, were analyzed using analysis of variances (ANOVA) with TukeyHSD post-hoc tests where applicable [58].

Principal Component Analysis (PCA), which reduces the dimensionality of data and identifies correlated variables, was used to separately analyze coral hosts and symbionts fatty acid composition for the full suite of 30 fatty acids as well as for PUFA [31,59,60]. For the PUFA analysis, the PCA included all (12) PUFA (18:2n-6, 18:3n-3, 18:3n-6, 18:4n-3, 20:2n-6, 20:3n-6, 20:4n-3, 20:4n-6, 20:5n-3, 22:4n-6, 22:5n-3, and 22:6n-3). Next, the compositions of metabolically important PUFA (18:2n-6, 18:3n-3, 18:3n-6, 18:4n-3, 18:5n-3, 20:4n-6, 20:5n-3, 22:6n-3) were compared among coral hosts, symbionts, and various potential prey sources including diatoms (n = 14), cyanobacteria (n = 11), dinoflagellates (n = 12), cryptophytes (n = 16), and tropical copepods (n = 19) (S3 Table). From a database of phytoplankton fatty acids, selected data i) were presented as proportions (percent of total fatty acids), ii) consisted of more than three individual fatty acids, iii) were of marine origin, iv) were not from polar regions, and v) were not subject to experimental treatments [29]. Phytoplankton were further filtered according to community composition recorded in Maldives reefs [61]. A linear discriminant analysis (LDA) (MASS package) was used to estimate the relative contribution of different sources of PUFA to each coral host species [62]. Using a leave-one-out cross-validation approach to evaluate the classification of potential sources of PUFA, sources with a classification rate <85% were removed from the analysis (S4 Table). The fatty acid 18:5n-3 was removed from the analysis because it was not present in the potential sources (copepods, diatoms, species-specific symbionts) used in the final model for each host species. A training data set was used to predict the contributions of various PUFA sources to coral host PUFA composition. A bootstrap approach was used to account for the within group variation in PUFA composition and to generate confidence estimates for the contribution of each group to coral host PUFA composition in the final model [63]. PUFA were re-sampled from each group (with replacement) and LDA was used to classify coral host samples of each species with each unique permutation of the raw data (n = 10,000). This approach maximized the variation in each putative source and generated a distribution of possible contributions to the diet of each coral species. This distribution of all possible diet combinations was used to determine the proportional contribution of particulate-derived versus symbiont-derived PUFA to each coral host species. To acknowledge that PUFA composition from “pure” autotrophy (only photosynthesis-derived) may differ from host-associated symbiont PUFA composition due to carbon cycling between coral hosts and their symbionts, an additional model including published data from cultured symbionts was considered [18]. Although the small sample size of cultured symbiont data precluded using LDA to classify coral host samples, the cultured symbiont samples appeared to comprise a separate group from the host-associated symbionts (S4 Fig). Therefore, results are discussed in terms of symbiont-derived PUFA rather than autotrophy-derived PUFA.

### 3.4 Ethics statement

Research was conducted under the Maldives Ministry of Fisheries and Agriculture permit (OTHR)30-D/INDIV/2016/556.

## 4. Results

### 4.1 Coral host and symbiont fatty acid compositions

**Fig 1:**
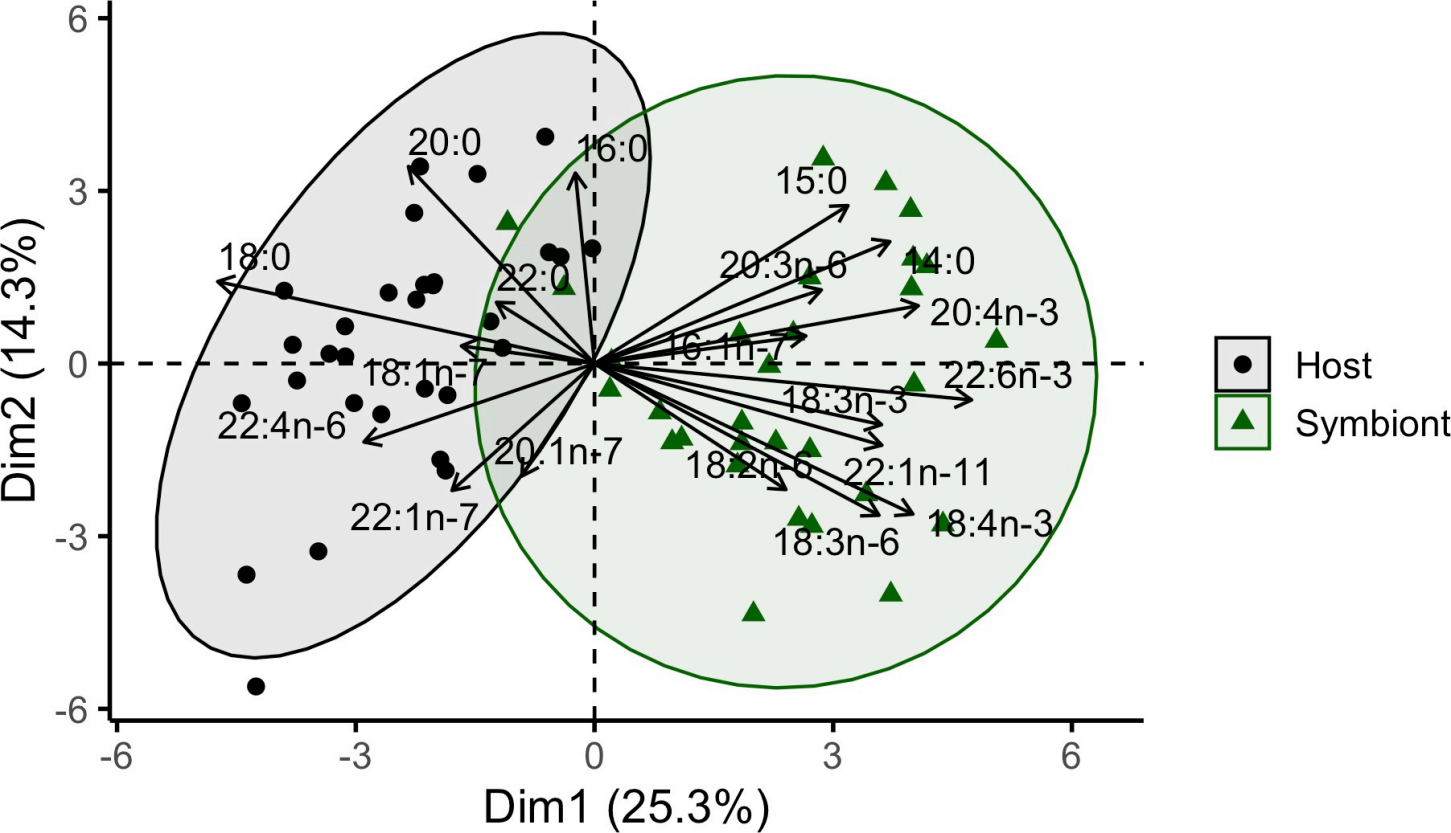
Total fatty acid composition by tissue type. Principal component analysis of the total fatty acid compositions of coral host species (*Galaxea fascicularis*, *Pachyseris speciosa*, *Pocillopora verrucosa*) and their symbionts with 95% confidence ellipses.

The composition of coral host and symbiont fatty acids were analyzed as proportions of total fatty acids (S1 Fig, S2 Table). PERMANOVA results revealed that depth was not a significant factor affecting coral host and symbiont fatty acid compositions (Pseudo-F_1,48_ = 0.687, P(perm) = 0.542). Therefore, data from both depths were combined for all analyses. Fatty acid composition was significantly affected by the interaction between tissue type (host versus symbiont) and the three coral species (Pseudo-F_2,48_ = 2.952, P(perm) = 0.019). PERMANOVA pairwise comparisons showed that the fatty acid composition of coral host and symbiont tissue were significantly different (P(perm)<0.001). For the fatty acid composition of host tissue, *P*. *speciosa* was significantly different than *P*. *verrucosa* (P(perm)<0.001) while *G*. *fascicularis* was significantly different than *P*. *speciosa* (P(perm) = 0.008) and *P*. *verrucosa* (P(perm) = 0.019). The fatty acid composition of *P*. *speciosa* symbionts was significantly different than *P*. *verrucosa* symbionts (P(perm) = 0.001) while *G*. *fascicularis* symbiont fatty acid composition was significantly different than that of *P*. *speciosa* symbionts (P(perm) = 0.020) and *P*. *verrucosa* symbionts (P(perm) = 0.021). Coral host samples clustered separately from symbiont samples in ordination space (Fig 1).

To investigate the similarity between host and symbiont fatty acid composition, the proportion of fatty acids were compared between coral host and symbiont tissue from individual coral colonies. The variation in host fatty acid proportions was reduced by considering symbiont proportions of the fatty acid, of which 14 individual fatty acids (comprising 47% of total fatty acids) had significant models (S2 Fig). The proportion of the MUFA 20:1n-9, a common marker for copepod feeding, showed a strong relationship (adj. R^2^ = 0.84) between *P*. *verrucosa* host and symbionts. The model of the Σ LC-MUFA index was also significant (S3 Fig). The saturated fatty acid 16:0 was the most prevalent fatty acid in both the hosts and symbionts, with means of 31.5–37.2% and 34.2–43.6%, respectively (S1 Fig, S2 Table). With a greater proportion in the different host species (means 22.2–29.0%) compared to symbiont tissues (means 7.0–12.9%), the fatty acid 18:0 appears to be an important component of storage and/or structural lipids in the coral hosts. The fatty acid 22:4n-6 was the third most dominant in the coral hosts (means 4.2–7.9%) but was found in lower proportions in the symbionts (means 1.3–2.8%). There was a significant interaction between Coral species and Tissue type for the fatty acid 22:4n-6 (ANOVA, F_2,54_ = 5.620, p = 0.006), which was greater in host rather than symbiont tissues for both *G*. *fascicularis* and *P*. *speciosa* (p<0.001). Further, the fatty acid 22:5n-3 was greater in host *P*. *verrucosa* than host *P*. *speciosa* (ANOVA, F_2,27_ = 5.861, p = 0.008; Tukey p = 0.006). The fatty acid 20:4n-6 was significantly higher in coral hosts than symbionts (ANOVA, F_2,54_ = 4.135, p = 0.047). The factors Coral species and Tissue type also had a significant effect on the fatty acid 18:3n-6 (ANOVA, F_2,54_ = 3.459, p = 0.039), with differences between *G*. *fascicularis* host and symbionts (p<0.001). Overall, *G*. *fascicularis* and *P*. *speciosa* symbionts had similar proportions of the fatty acid 18:3n-6 (p = 0.190). The fatty acids 18:3n-6 (4.7–8.7%) and 18:4n-3 (5.2–6.9%) were higher in symbionts than in the coral hosts, in which the sum of both fatty acids was <3.5%. Very low proportions of 18:3n-3 were found in the symbionts (mean <0.1%) and this fatty acid was not found in any host samples. In addition to analyzing the proportion of fatty acids, the mass of total fatty acids per tissue type of each coral species was quantified. Symbionts had considerably higher mass (8–16 fold) of total fatty acids compared to their coral hosts (S2 Table).

### 4.2 Composition of polyunsaturated fatty acids (PUFA)

**Fig 2:**
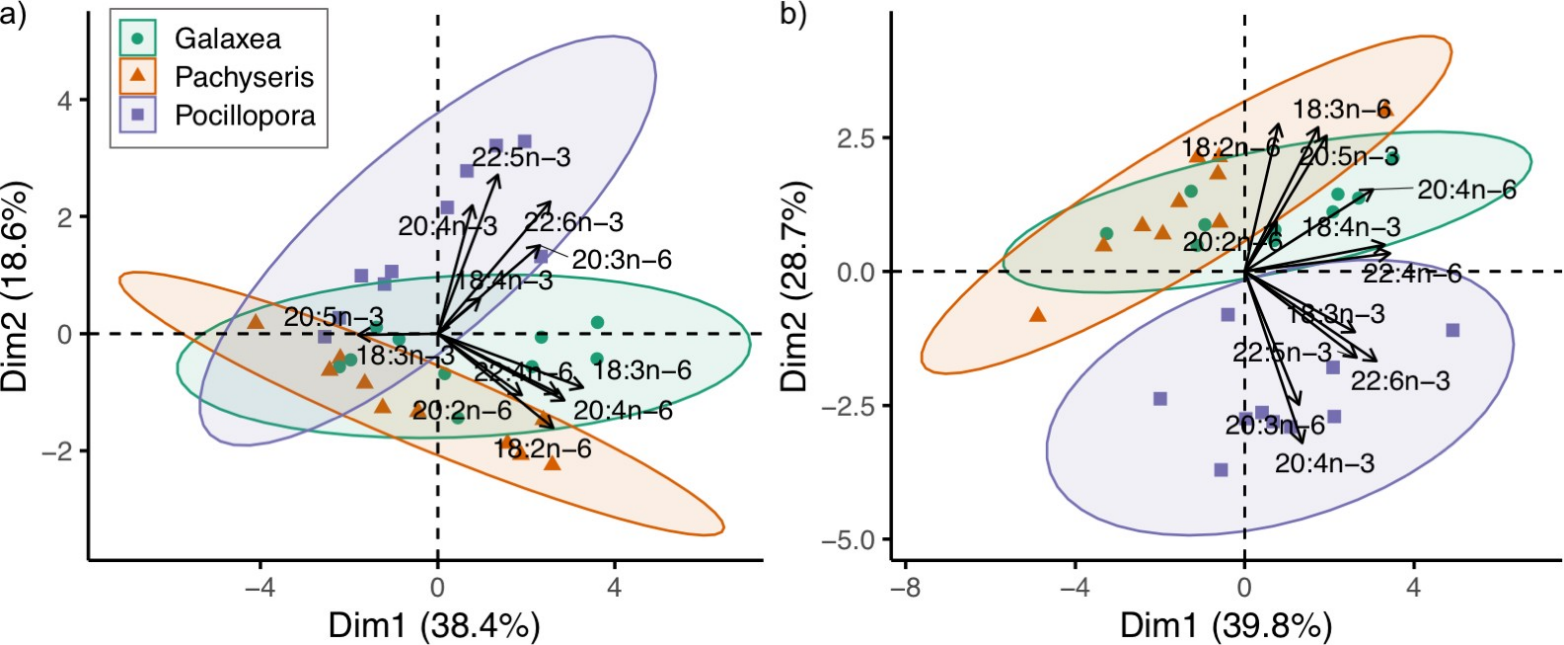
Comparison of polyunsaturated fatty acid (PUFA) composition among coral host species and associated symbionts. Principal component analysis based on the PUFA composition of *Galaxea fascicularis*, *Pachyseris speciosa*, and *Pocillopora verrucosa* a) coral hosts and b) their associated symbionts with 95% concentration ellipses.

A PCA was used to examine the PUFA composition of the coral hosts and symbionts (all 12 PUFA, S2 Table), with most important variables listed in order of higher to lower contribution. The 95% confidence interval ellipses showed separation of host *P*. *verrucosa* from host *P*. *speciosa* and host *G*. *fascicularis* (Fig 2A). Principal component Dim1 explained 38.4% of the host PUFA variability, with a majority of the contribution from n-6 PUFA (18:3n-6, 20:4n-6, 22:4n-6, 18:2n-6). Three n-3 PUFAs (22:5n-3, 22:6n-3, 20:4n-3) contributed the most to principal component Dim2, which explained 18.6% of the variability in the host PUFA composition. The fatty acid 20:5n-3 contributed to the similarity among the species along the Dim1 axis. In the PCA of symbiont PUFA, *P*. *verrucosa* symbionts were distinctly separated from *P*. *speciosa* symbionts and *G*. *fascicularis* symbionts, of which the latter two showed some overlap in their ellipses (Fig 2B). Both n-3 and n-6 PUFA (22:4n-6, 18:4n-3, 22:6n-3, 20:4n-6, 22:5n-3, 18:3n-3) contributed to the principal component Dim1, which explained 39.9% of the variability in symbiont PUFA composition. Symbiont PUFA composition was also explained by the principal component Dim2 (28.7%), with contributions from the fatty acids 20:4n-3, 18:2n-6, 18:3n-6, 20:5n-3, and 20:3n-6.

### 4.3 Evaluating coral trophic strategies using fatty acid biomarker indices

**Fig 3:**
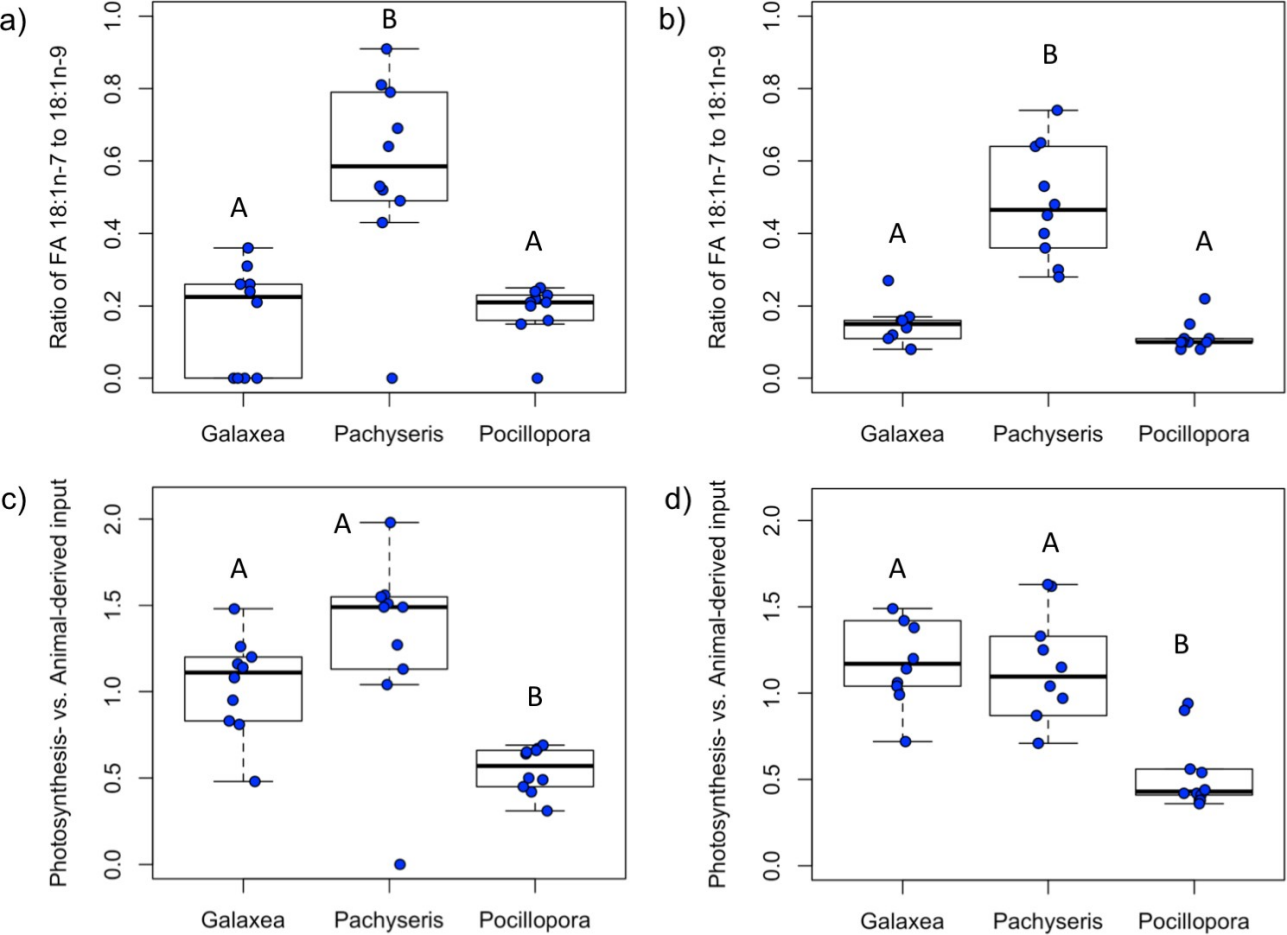
Fatty acid indices in the context of coral trophic strategies. Established fatty acid indices are applied in a novel context as potential indicators of coral trophic strategies (autotrophy versus heterotrophy). The ratio of fatty acids 18:1n-7 to 18:1n-9 shows the relative proportion of photosynthesis-derived versus animal-derived nutrition for corals *Galaxea fascicularis*, *Pachyseris speciosa*, and *Pocillopora verrucosa* in their a) host tissues and b) symbiont tissues. An additional index of photosynthesis- versus animal-derived nutrition considers the input of typically photosynthesis-derived fatty acids (16:1n-7 and 18:1n-7) relative to typically animal-derived fatty acids (18:1n-9, 20:1n-9, and 22:1n-11) for c) coral host tissues and d) symbiont tissues. Statistical differences are designated within each boxplot with capital letters.

An indicator of the degree of photosynthesis- versus animal-derived nutrient input (i.e., ratio of 18:1n-7 to 18:1n-9) showed a higher ratio in the host and symbionts of *P*. *speciosa* compared to the host and symbionts of *P*. *verrucosa* and *G*. *fascicularis* (Fig 3). For the ratio of fatty acids 18:1n-7 to 18:1n-9, there were significant differences among the coral host species (ANOVA, F_2,27_ = 17.74, p<0.001). Pairwise comparisons showed that host *P*. *speciosa* had significantly higher 18:1n-7 to 18:1n-9 ratios than host *G*. *fascicularis* (p<0.001) and host *P*. *verrucosa* (p<0.001; Fig 3A). Similarly, there were significant differences in the ratio of 18:1n-7 to 18:1n-9 among the symbionts (ANOVA, F_2,27_ = 43.8, p<0.001) with a higher ratio in *P*. *speciosa* symbionts compared to *G*. *fascicularis* symbionts (p<0.001) and *P*. *verrucosa* symbionts (p<0.001; Fig 3B).

A related index of photosynthesis- (16:1n-7 and 18:1n-7) vs. animal-derived dietary input (18:1n-9, 20:1n-9, and 22:1n-11) differed among coral hosts (ANOVA, F_2,27_ = 11.76, p<0.001), with a lower ratio in host *P*. *verrucosa* compared to host *P*. *speciosa* (p<0.001) and host *G*. *fascicularis* (p = 0.012; Fig 3C). The photosynthesis- vs. animal-derived dietary input was also different among symbionts (ANOVA, F_2,27_ = 11.76, p<0.001), with a higher ratio in *G*. *fascicularis* symbionts (p<0.001) and *P*. *speciosa* symbionts (p = 0.003) compared to *P*. *verrucosa* symbionts (Fig 3D).

**Fig 4:**
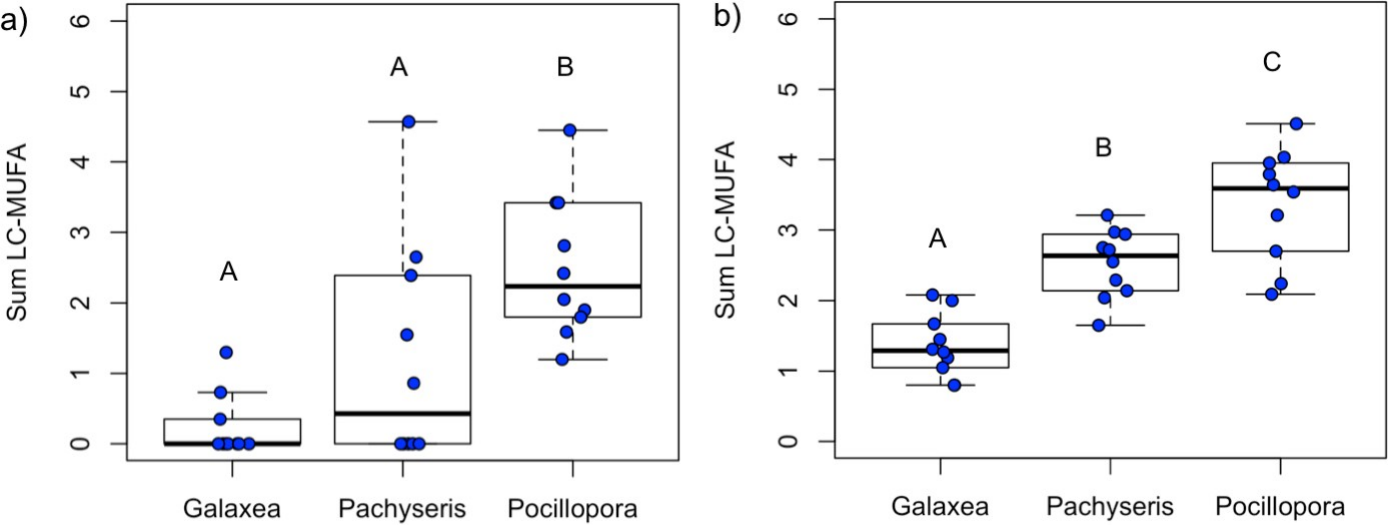
Sum of long-chain monounsaturated fatty acids (LC-MUFA). The fatty acid index of the sum of LC-MUFA (Σ20:1 and Σ22:1, as percent of total fatty acids) was used to evaluate the relative proportion of nutrition derived from copepods for the corals *Galaxea fascicularis*, *Pachyseris speciosa*, and *Pocillopora verrucosa* in their a) host tissues and b) symbiont tissues. Statistical differences are designated within each boxplot with capital letters.

The effect of Coral species and Tissue type were tested by evaluating the sum of LC-MUFA as an index of potential heterotrophic feeding on copepods. Coral species affected the sum of the LC-MUFA among the coral hosts (ANOVA, F_2,27_ = 10.52, p<0.001) with host *P*. *verrucosa* having a higher proportion of the ΣLC-MUFA than host *G*. *fascicularis* (p<0.001) and host *P*. *speciosa* (p = 0.036) (Fig 4A). The proportion of the ΣLC-MUFA was the most variable in host *P*. *speciosa*. In symbionts, coral species also affected the proportion of ΣLC-MUFA (ANOVA, F_2,27_ = 28.35, p<0.001). The proportion of the ΣLC-MUFA in *P*. *verrucosa* symbionts was greater than *P*. *speciosa* symbionts (p = 0.011) and *G*. *fascicularis* symbionts (p<0.001) (Fig 4B). Further, the proportion of the ΣLC-MUFA was greater in *P*. *speciosa* symbionts than *G*. *fascicularis* symbionts (p<0.001).

### 4.4 Estimating symbiont- and particulate-derived PUFA in coral hosts

**Fig 5:**
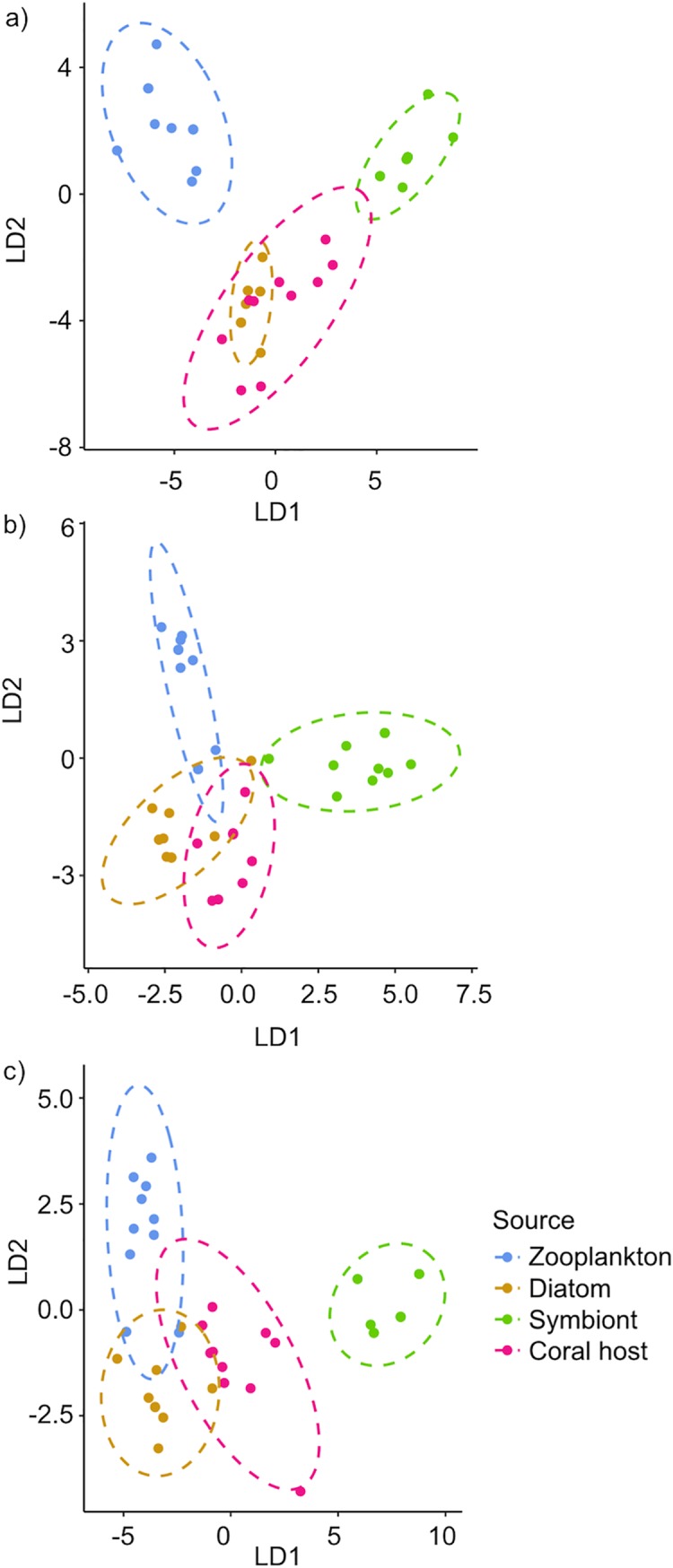
Linear discriminant analysis of polyunsaturated fatty acids (PUFA). Linear discriminant analysis based on PUFA composition (18:2n-6, 18:3n-3, 18:3n-6, 18:4n-3, 20:4n-6, 20:5n-3, 22:6n-3) was used to estimate the proportional contribution of different sources of PUFA to the host PUFA composition of corals a) *Galaxea fascicularis*, b) *Pachyseris speciosa*, and c) *Pocillopora verrucosa* (95% confidence ellipses).

A linear discriminant analysis was used to estimate the proportion of coral PUFA derived from various potential sources including symbionts and various particulate prey items including copepods and diatoms (Fig 5). Overall, 95% confidence intervals indicated that species-specific symbiont PUFA has a lower contribution (0–40%) to coral host PUFA for both *G*. *fascicularis* and *P*. *verrucosa* while the contribution of symbiont-derived PUFA was variable but may be more important (0–62%) for coral host *P*. *speciosa*. Particulate resources are likely an important source of PUFA for all coral hosts especially *G*. *fascicularis* and *P*. *verrucosa* (60–100%) as indicated by 95% confidence intervals. Between the particulate prey sources examined in the final model, copepod-derived PUFA was more important (mean 12 ± 5%) for host *P*. *verrucosa* compared to the other species (mean 1 ± 1%) while diatom-derived PUFA represented an important contribution to all three host species (mean 87%).

## 5. Discussion

Although the mixotrophic nature of symbiotic reef-building corals enables them to live across a variety of environmental conditions, the complexity of symbiosis poses challenges to deciphering the trophic strategies of reef-building corals. Indeed, symbiont type can influence host metabolism and trophic plasticity [64,65]. Biochemical tracers such as fatty acids are key to studying trophic ecology because multiple tracers can provide insight into the origin of dietary resources. Despite the difference in the total mass of fatty acids between coral hosts and symbionts, there were significant relationships in the proportions of 14 individual fatty acids (comprising 47% of total fatty acids). Although early studies showed that fatty acids were synthesized by symbionts and subsequently transferred to the coral host [66], the bidirectional interchange of fatty acids between a coral host and its symbionts is possible given that both partners can synthesize PUFA *de novo* [22] and/or obtain nutrients from the diet [5,19], with hosts capable of transferring PUFA to symbionts [27]. Our study used several approaches to assess the contribution of host heterotrophy and symbiont autotrophy to coral fatty acid composition.

### 5.1 Coral trophic strategies as defined by fatty acids

The ratio of 18:1n-7 to 18:1n-9 fatty acids, which can reflect photosynthesis- versus animal-derived input, showed patterns that reflect differences in coral trophic strategies (Fig 3). The lower ratios of 18:1n-7 to 18:1n-9 fatty acids in *P*. *verrucosa* and *G*. *fascicularis* corals are consistent with a greater contribution from heterotrophic feeding, which is supported by carbon stable isotopes and experimental feeding studies [50,67,68]. Heterotrophic feeding by *G*. *fascicularis* can support symbiont autotrophy [52,69], which is reflected by the different responses of *G*. *fascicularis* for the two related fatty acid indices (Fig 3). The more heterotrophic coral, *P*. *verrucosa*, had a lower index of photosynthesis- vs. animal-derived dietary input compared to the more autotrophic corals *P*. *speciosa* and *G*. *fascicularis*. As the fatty acid 22:5n-3 is typically found in higher proportions in asymbiotic corals (without photosynthetic symbionts) that exclusively rely on heterotrophic feeding [32,70], a higher proportion of 22:5n-3 indicates greater heterotrophy in host *P*. *verrucosa* compared to host *P*. *speciosa*. The coral *P*. *speciosa* had the highest ratio of 18:1n-7 to 18:1n-9, indicating a greater contribution from photosynthesis-derived products that may be due to higher symbiont autotrophy and/or nutrients from feeding on phytoplankton. Carbon stable isotope ratios and efficient photosynthesis across different light regimes have shown that autotrophy is the primary trophic strategy of *P*. *speciosa* [50,71,72]. Future directions include tracing the origin and relative proportion of energy from different sources [73], such as employing isotopic labeling to trace fatty acids from diverse potential prey.

Several lines of evidence provide support for heterotrophic feeding by *P*. *verrucosa*, which can capture prey with its polyps using nematocysts as well as through extruding a mucus web [67]. The sum of the LC-MUFA index indicated that host *P*. *verrucosa* had the highest proportion of fatty acids potentially derived from copepods, which is similar to previous results for this species from other reef systems [42]. In particular, there was a strong relationship in the proportion of the common marker for copepod feeding (20:1n-9) between *P*. *verrucosa* host and symbionts (S2H Fig). Further, the LDA model estimated that the mean PUFA contribution from copepods was greatest for the coral host *P*. *verrucosa* (12%) compared to the other coral species (1%). The convergence of MUFA and PUFA results provide strong support for *P*. *verrucosa* heterotrophic feeding. Indeed, pocilloporid corals consume a variety of taxa including copepods, microalgae, planktonic larvae, and rotifers [67,74] and water column primary productivity is an important driver of heterotrophy for *Pocillopora* species [75]. Despite the importance of plankton as a food source for marine fauna in the Maldives including corals and various megafauna [76], limited information is available about the plankton community in reef waters of the central Indian Ocean [77]. There is a clear need for research on plankton community composition and abundance throughout the reef water column across the Maldives archipelago.

Although PUFA composition is often used for coral chemotaxonomy [31,70], the application of PUFA composition in characterizing coral trophic strategies has yet to be addressed [28]. For coral hosts, n-3 PUFA contributed to the different clustering of the more heterotrophic *P*. *verrucosa* while n-6 PUFA drove the separation of the more autotrophic *P*. *speciosa* (Fig 2). Symbionts of *P*. *verrucosa* consisted of a distinct group in contrast to the clustering of *P*. *speciosa* and *G*. *fascicularis* symbionts (Fig 2). Symbionts are considered to be the primary source of 18:3n-6, which is found in higher proportions in symbionts than the host tissue [17,27]. In this study, the fatty acid 18:3n-6 was significantly higher in symbionts particularly in the more autotrophic corals *G*. *fascicularis* and *P*. *speciosa* (S2F Fig). Relatively higher proportions of 18:2n-6 in symbionts of the more autotrophic corals *P*. *speciosa* and *G*. *fascicularis* may indicate greater symbiont autotrophy compared to *P*. *verrucosa* symbionts as previous work has shown lower proportions of this fatty acid in symbionts under low light conditions [78]. In contrast, proportions of the longer chain n-6 PUFA (i.e., 20:4n-6 and 22:4n-6) were significantly higher in host tissue. Experimental evidence for host synthesis of fatty acid 22:4n-6 has been demonstrated in symbiotic anemones [25]. The fatty acid 22:4n-6 is typically abundant in coral host tissue [12,31,78], and has been suggested as a marker of the coral host [27].

### 5.2 Contribution of PUFA sources to coral host composition

To evaluate the extent of autotrophy and heterotrophy in corals, it is necessary to consider potential sources of fatty acids. Overall, each species of coral host showed a distinct composition of PUFA compared to likely sources of potential prey. PUFA derived from particulate resources was estimated to provide a greater contribution (60–100%) than symbiont-derived PUFA (0–40%) to host PUFA composition of *P*. *verrucosa* and *G*. *fascicularis* in particular. Indeed, previous work has shown a similar importance of heterotrophic carbon to coral nutrition [8,73,79]. Among the three species of coral hosts, a majority of PUFA (mean 87%) appeared to be derived from diatoms. The larger estimated contribution from diatom-derived fatty acids rather than copepod-derived fatty acids aligns with some of the passive feeding strategies exhibited by the corals (e.g., mucus webs, extracoelenteric feeding, mesenterial feeding) [74,80,81]. Despite the limited information on phytoplankton communities in tropical Indian Ocean reef waters, diatoms are among the dominant taxa recorded [61,82]. Phytoplankton can be an important source of nutrients for reef ecosystems [83,84]. Notably, diatom depletion can be greater than zooplankton depletion in some reef systems [85] and diatom-derived organic matter can be a source of nutrients for coral reef organisms such as sponges [86]. Although zooplankton have been typically considered the primary particulate food source for reef-building corals [2], the extent to which phytoplankton may contribute to coral nutrition remains poorly understood [87,88]. The fatty acid analyses in this study highlight the potential importance of phytoplankton, a source often overlooked in examining coral diets.

Our study demonstrates that the fatty acid composition of corals can be used to understand the trophic strategies of reef-building corals, with the fatty acid results aligning closely with previous carbon stable isotope results for the same three species [50]. Eight months following a thermal stress event in the Maldives, this study shows that PUFA from particulate sources appear to be a major resource for corals in particular *G*. *fascicularis* and *P*. *verrucosa*. High primary productivity supported by deep-water upwelling sustains relatively high coral heterotrophy in the Maldives relative to corals from less productive reef systems [75]. Whether coral heterotrophy reflects the status quo supported by the productive waters of the Maldives as opposed to a prolonged stress response (i.e., heterotrophic compensation) requires the regular monitoring of coral and symbiont fatty acid composition over time [89]. Mixotrophic organisms may become more heterotrophic under conditions of rising temperatures, but it is unclear if such changes in trophic behavior indicate underlying physiological stress as opposed to a measure of resilience [89,90]. Besides the general importance of heterotrophy in mixotrophic organisms, the ability of corals to obtain nutrients from different resources is critical when the symbiosis and the presumably major autotrophic energy source is compromised [79,91]. Our finding that symbionts have a greater mass of total fatty acids than coral host tissues is similar to previous work [17,92], and also is consistent with the potential depletion of host lipids following the 2016 global thermal stress event that caused mass coral bleaching across the Maldives [93,94]. Although thermal stress conditions can affect coral fatty acid composition and the expression of symbiont fatty acid desaturases [95,96], heterotrophic feeding can lessen the potential impact of thermal stress conditions on coral fatty acids [11]. In particular, feeding on PUFA-enriched particulate food sources can mitigate coral bleaching under thermal stress conditions [34]. Future work should investigate the potential effect of a trophic enrichment factor in the assimilation of fatty acids (host particulate feeding) as well as the synthesis and transfer of fatty acids between coral hosts and their symbionts under normal and thermal stress conditions. Given the frequency of thermal stress events that cause coral bleaching, it is especially important to characterize coral trophic strategies and investigate the mechanisms by which corals survive and recover as species-specific trophic strategies may underlie shifts in coral communities [97].

## Supporting information

S1 Table Collection sites. Geographic coordinates.
(XLSX)

S2 Table Raw data total fatty acid composition. Fatty acid composition (mean ± SD of % total fatty acids) of three species of coral hosts and their symbionts from the Maldives (n = 10 samples for each species of host or symbiont).
(XLSX)

S3 Table Raw data polyunsaturated fatty acids (PUFA). Polyunsaturated fatty acids of coral hosts, their symbionts, and various potential prey sources obtained from the literature.
(XLSX)

S4 Table Linear discriminant analysis source classification. Classification of different potential sources of polyunsaturated fatty acids (18:2n-6, 18:3n-6, 18:3n-3, 18:4n-3, 18:5n-3, 20:4n-6, 20:5n-3, 22:6n-3) evaluated in the original model of the linear discriminant analysis. Species-specific symbionts were evaluated for each coral host model.
(XLSX)

S1 Fig Proportional differences in coral host and symbiont fatty acids. Comparison of the proportional difference of thirty fatty acids between coral hosts (red) and symbionts (green) for three species of corals a) *Galaxea fascicularis*, b) *Pachyseris speciosa*, and c) *Pocillopora verrucosa*. Number inside colored circle is the difference between coral host and symbiont fatty acid percentage, with color and x-axis position relative to zero signifying which partner has a greater proportion of a given fatty acid. A blank space is shown if there was zero percent of a given fatty acid. A negative number of host fatty acid percent difference is due to the position (left) in relation to zero on the x-axis.
(TIFF)

S2 Fig Relationship between coral host and symbiont fatty acid proportions. The relationship between symbiont and host fatty acid proportions of fatty acids (a-n), from individual coral colonies of *Galaxea fascicularis* (red), *Pachyseris speciosa* (green), and *Pocillopora verrucosa* (blue).
(PDF)

S3 Fig Relationship between the proportions of coral host and symbiont long-chain monounsaturated fatty acids. Proportion of the sum of long-chain monounsaturated fatty acids (LC-MUFA) between symbionts and hosts of individual colonies of the corals *Galaxea fascicularis* (red), *Pachyseris speciosa* (green), and *Pocillopora verrucosa* (blue).
(TIFF)

S4 Fig Linear discriminant analysis considering cultured symbionts. Linear discriminant analysis (LDA) based on the polyunsaturated fatty acid composition (18:2n-6, 18:3n-3, 18:3n-6, 18:4n-3, 20:4n-6, 20:5n-3, 22:6n-3) of coral hosts, most likely potential food sources (as per final LDA model), host-associated symbionts, and cultured symbionts. Despite the low sample size of cultured symbiont fatty acid data available in the literature, the cultured symbiont samples group closely together and do not clearly cluster with host-associated symbionts.
(TIFF)
