# Supplementary figures and images for: Evaluating coral trophic strategies using fatty acid composition and indices
Source: PLoS One. 2019 Sep 11;14(9):e0222327. doi: 10.1371/journal.pone.0222327 (PMC6739055; doi:10.1371/journal.pone.0222327)

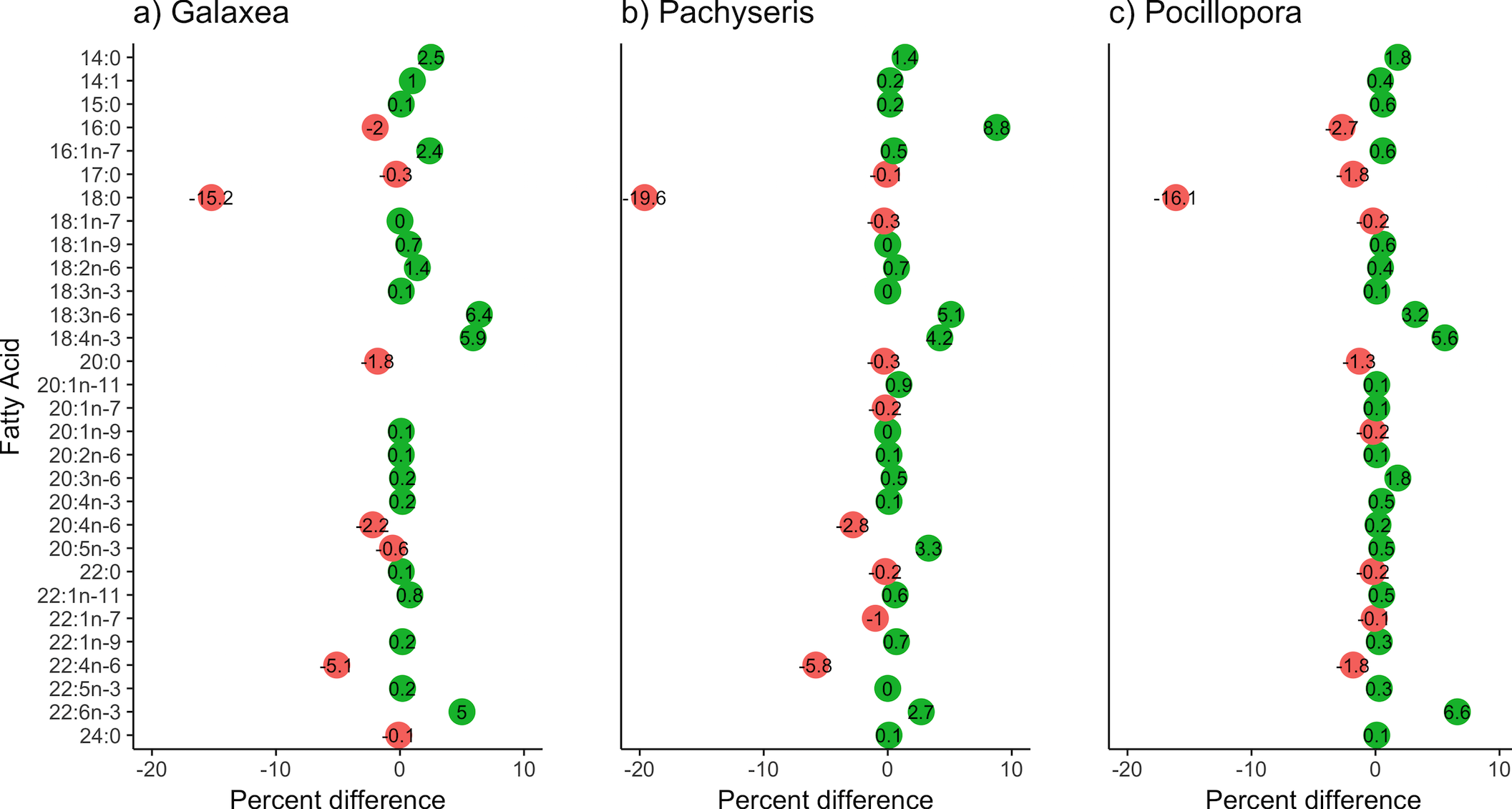

Supplement: S1 Fig — Comparison of the proportional difference of thirty fatty acids between coral hosts (red) and symbionts (green) for three species of corals a) Galaxea fascicularis, b) Pachyseris speciosa, and c) Pocillopora verrucosa. Number inside colored circle is the difference between coral host and symbiont fatty acid percentage, with color and x-axis position relative to zero signifying which partner has a greater proportion of a given fatty acid. A blank space is shown if there was zero percent of a given fatty acid. A negative number of host fatty acid percent difference is due to the position (left) in relation to zero on the x-axis. (TIFF) [file pone.0222327.s005.tiff]

## S2 Figure

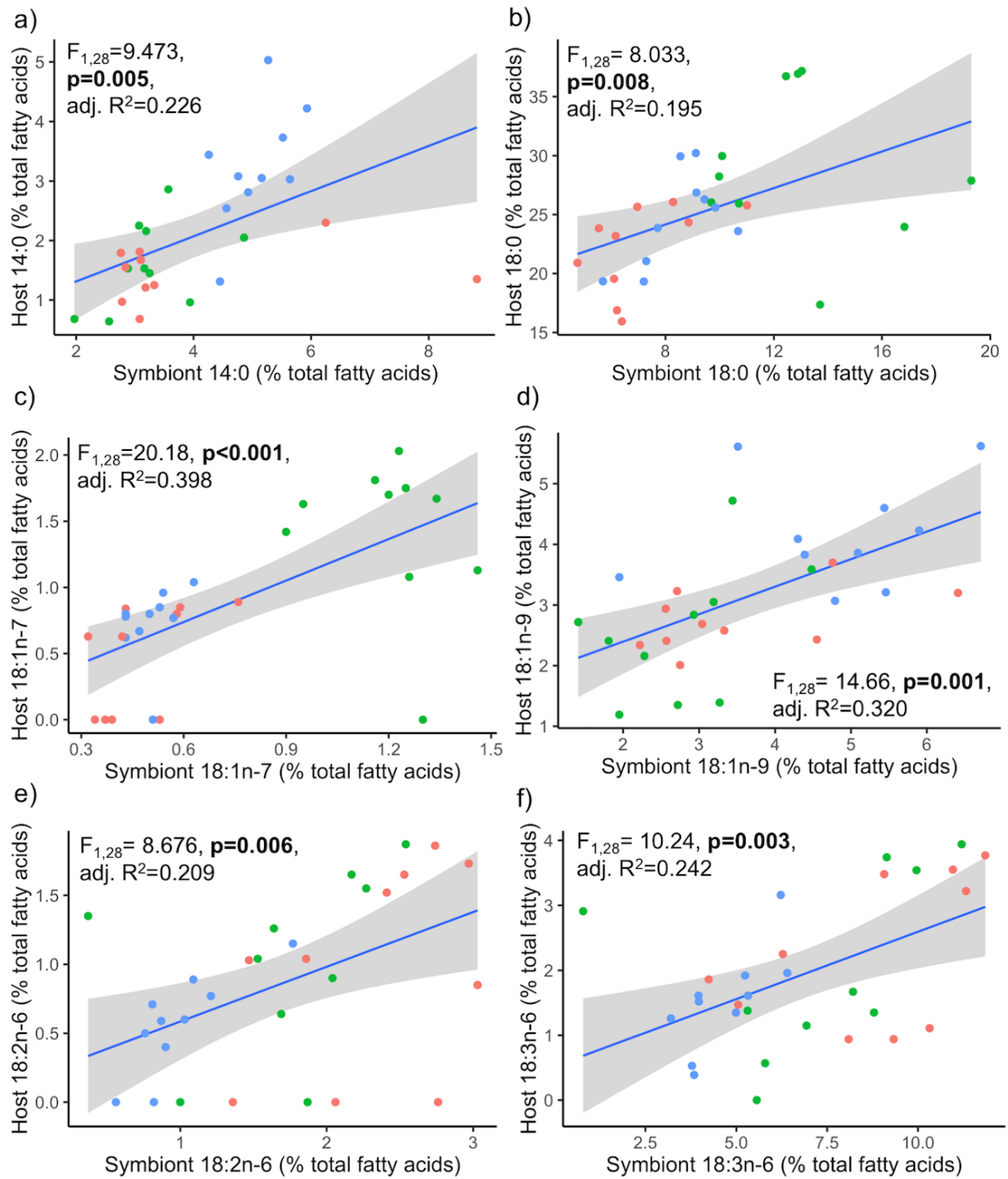

g)

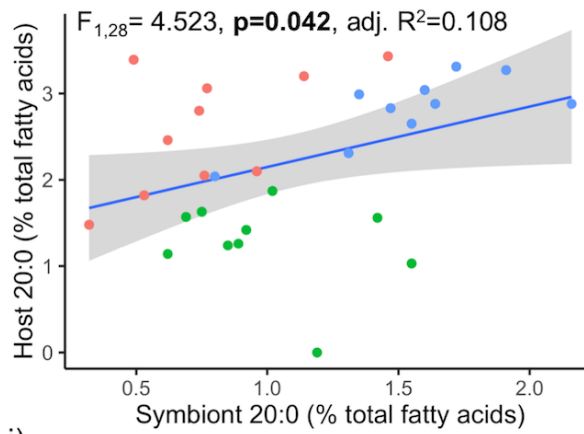

h)

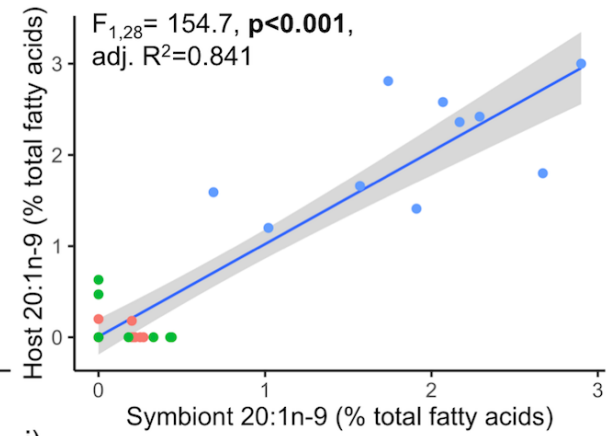

i)

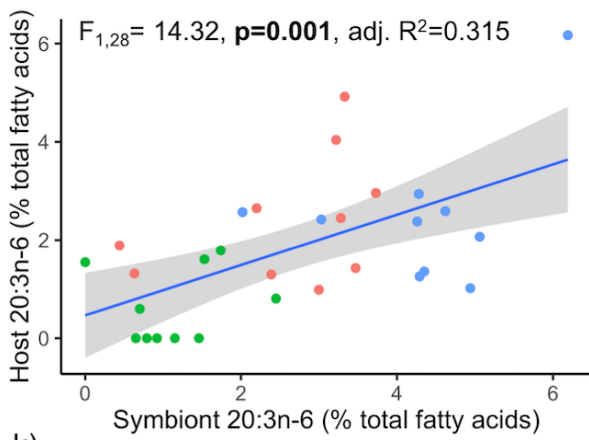

j)

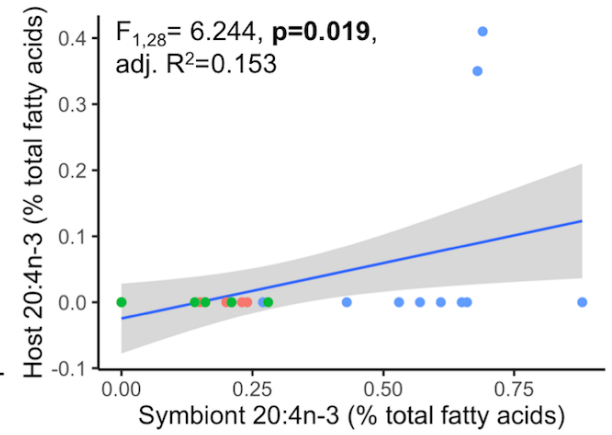

k)

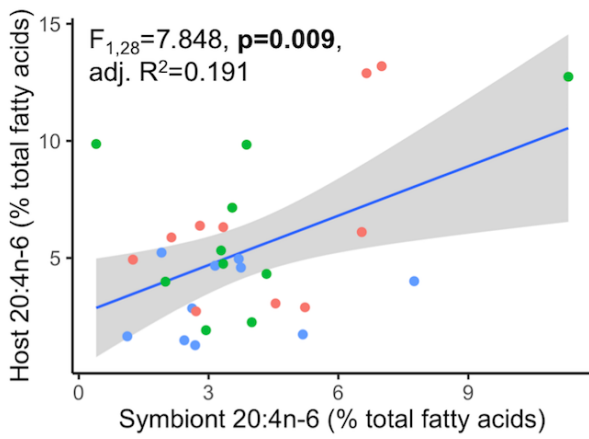

l)

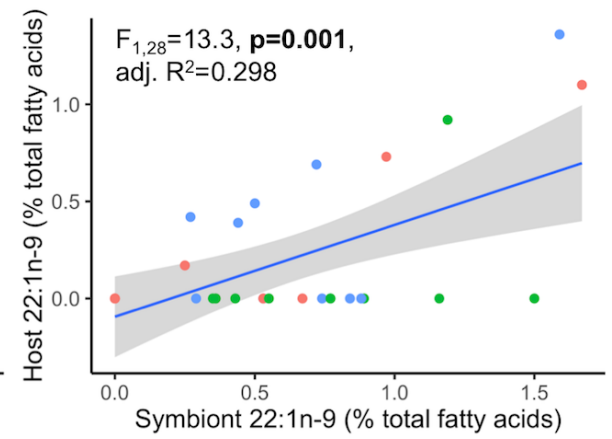

m)

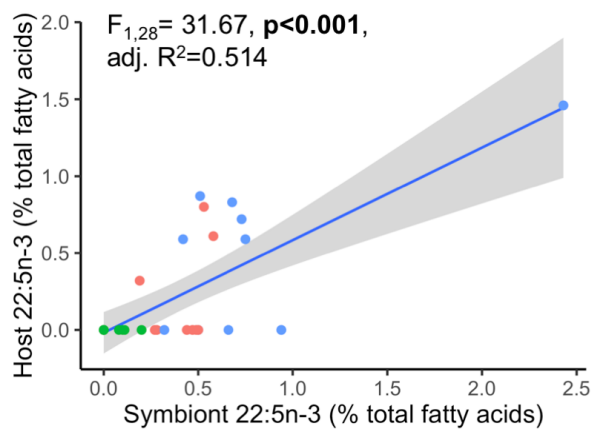

n)

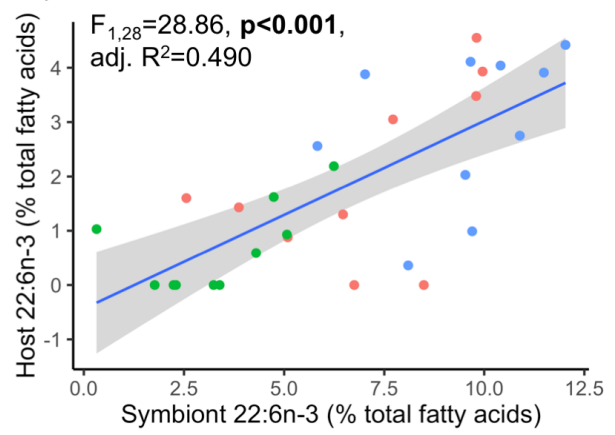

Supplement: S2 Fig — The relationship between symbiont and host fatty acid proportions of fatty acids (a-n), from individual coral colonies of Galaxea fascicularis (red), Pachyseris speciosa (green), and Pocillopora verrucosa (blue). (PDF) [file pone.0222327.s006.pdf]

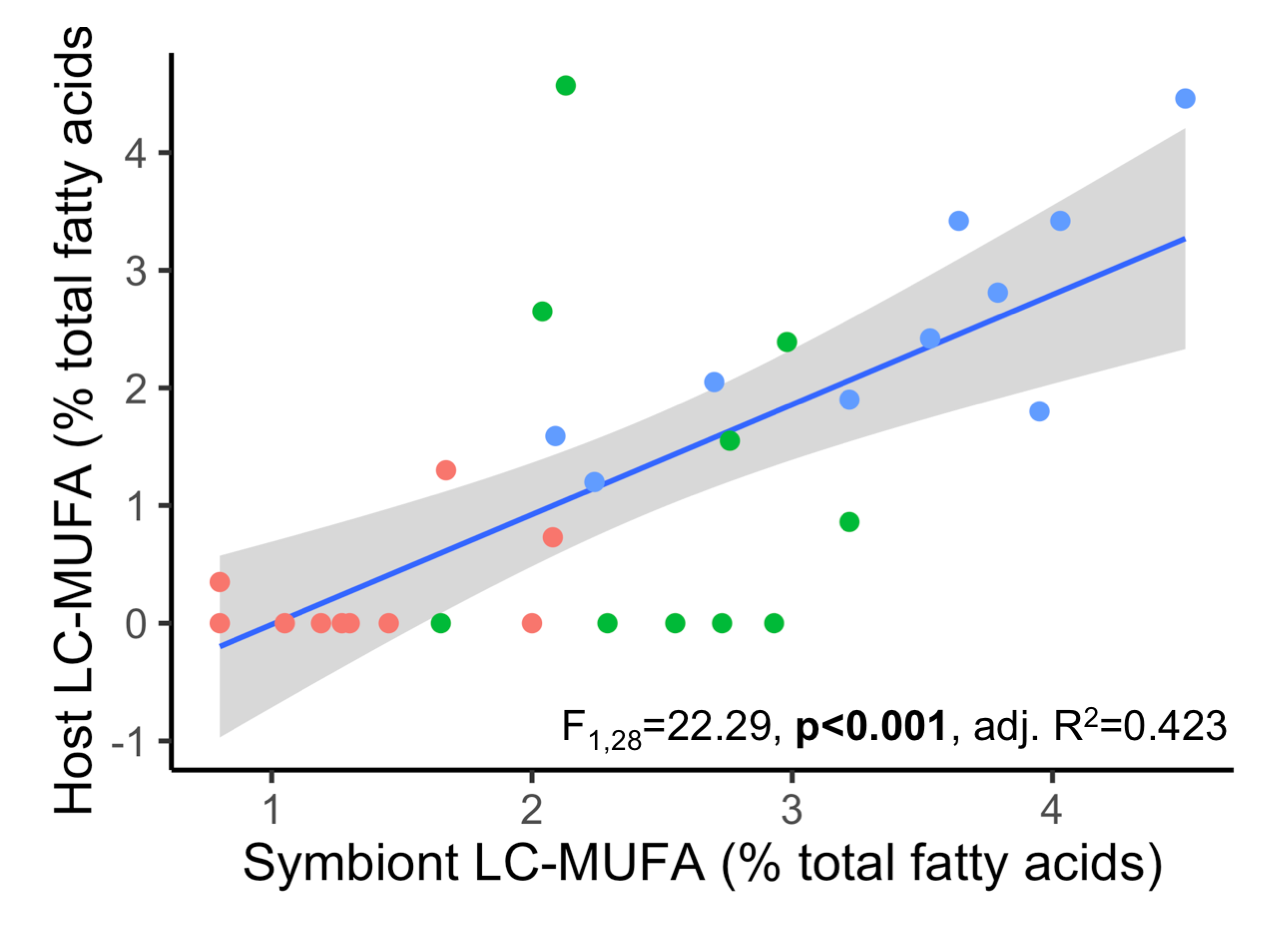

Supplement: S3 Fig — Proportion of the sum of long-chain monounsaturated fatty acids (LC-MUFA) between symbionts and hosts of individual colonies of the corals Galaxea fascicularis (red), Pachyseris speciosa (green), and Pocillopora verrucosa (blue). (TIFF) [file pone.0222327.s007.tiff]

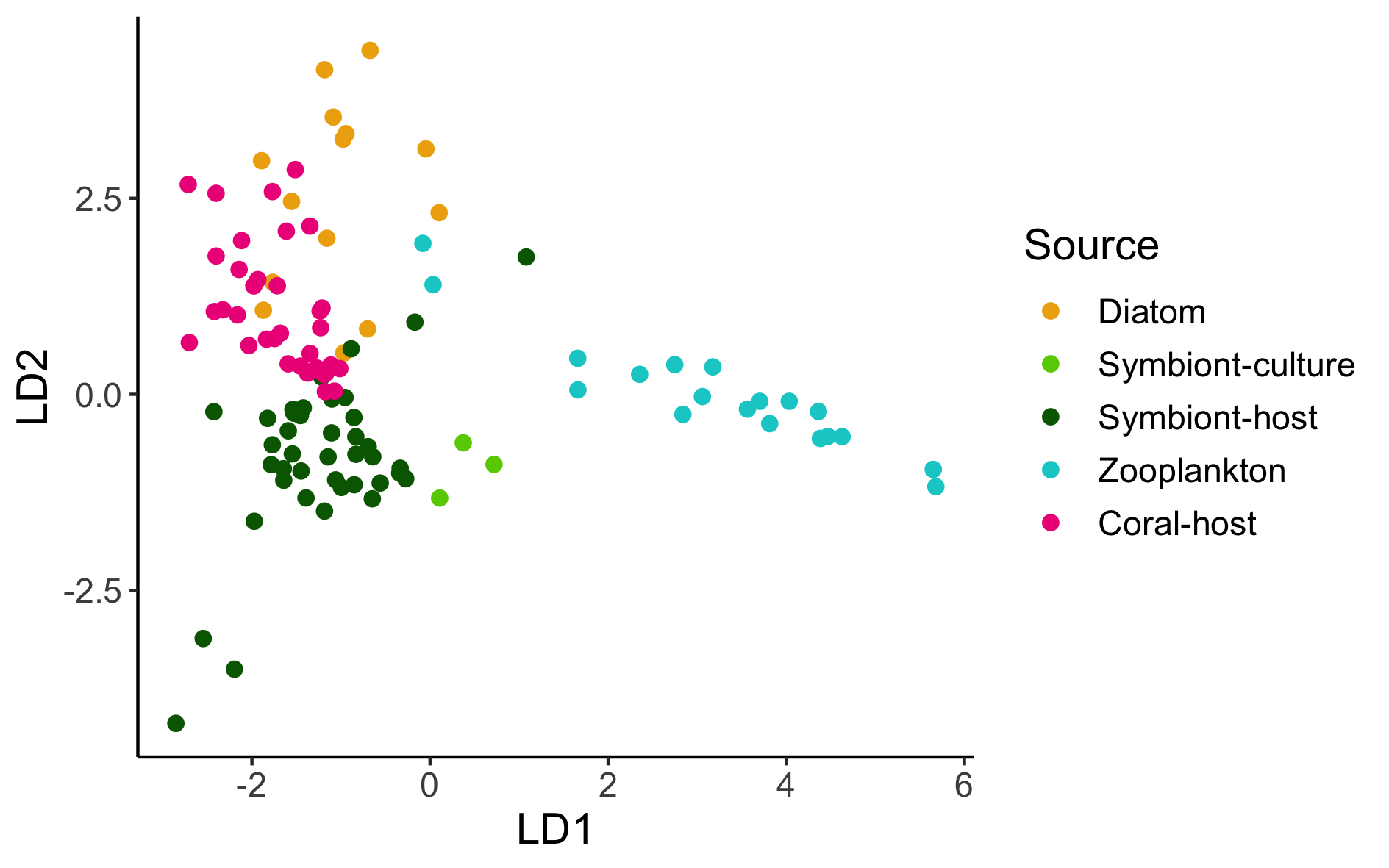

Supplement: S4 Fig — Linear discriminant analysis (LDA) based on the polyunsaturated fatty acid composition (18:2n-6, 18:3n-3, 18:3n-6, 18:4n-3, 20:4n-6, 20:5n-3, 22:6n-3) of coral hosts, most likely potential food sources (as per final LDA model), host-associated symbionts, and cultured symbionts. Despite the low sample size of cultured symbiont fatty acid data available in the literature, the cultured symbiont samples group closely together and do not clearly cluster with host-associated symbionts. (TIFF) [file pone.0222327.s008.tiff]
